# Mapping psychosocial interventions in familial colorectal cancer: a rapid systematic review

**DOI:** 10.1186/s12885-021-09086-8

**Published:** 2022-01-03

**Authors:** Andrada Ciucă, Ramona Moldovan, Adriana Băban

**Affiliations:** 1 Department of Psychology, Babeş-Bolyai University, Cluj-Napoca, Romania; 2 Division of Evolution and Genomic Sciences, School of Biological Science, University of Manchester, Manchester, UK; 3 Manchester Centre for Genomic Medicine, St Mary’s Hospital, Manchester University Hospitals NHS Foundation Trust, Manchester, UK

**Keywords:** Psychosocial interventions, Genetic counselling, Familial colorectal cancer, Systematic review

## Abstract

**Background:**

Approximately 5% of colorectal cancer (CRC) cases are part of a well-defined inherited genetic syndrome and up to approximately 30% of these cases have a clinically defined familial basis. Psychosocial interventions in familial colorectal cancer address aspects mainly focused on affective, cognitive and behavioural outcomes. The present review aims to systematically map out the available psychosocial interventions for individuals with a family history of CRC and describe the current state of the research.

**Methods:**

An extensive electronic search was conducted to investigate the literature published until June 2020. Inclusion criteria consisted of quantitative studies published in English that explored the impact of psychosocial interventions for familial CRC, clearly defined the psychosocial intervention offered and included participants with a family history of CRC.

**Results:**

The analysis included 52 articles. Genetic counselling, educational interventions, psychological interventions and multimodal interventions were identified across the studies. In terms of diagnoses, Lynch Syndrome, Familial Adenomatous Polyposis, Familial Colorectal Cancer were the main conditions included in the studies. Affective, cognitive, behavioural aspects and quality of life emerged as the most frequently explored outcomes. The studies included individuals with both personal and familial history of CRC or family history alone.

**Conclusions:**

Our rapid review provides an overview of the literature exploring the impact of psychosocial interventions for familial CRC. The psychosocial interventions identified had an overwhelmingly positive impact across all types of outcomes measured. Genetic counselling appeared to be most beneficial, and this is expected as it is purposively designed to address genetic conditions. Further quantitative analysis of primary empirical research is needed to determine the efficacy and effectiveness of psychosocial interventions as well as the mechanisms through which they exert their effect.

## Introduction

Colorectal cancer (CRC) is the third most frequent form of cancer and the third leading cause of cancer death [1]. A family history of CRC is known to be associated with an increased risk of developing CRC [2]. Approximately 5% of CRC cases are part of a well-defined inherited genetic syndrome [3] such as Lynch Syndrome (LS) and Familial Adenomatous Polyposis (FAP). Also, up to approximately 30% of the total cases of CRC have a clinically defined familial basis [3] and, for the purpose of this review, are clustered under the familial colorectal cancer label (fCRC).

Psychosocial interventions address various psychological and social aspects of a condition and can be delivered in a counselling format, as health education or with a focus on social support. In familial CRC, psychosocial interventions are usually focused on (1) affective outcomes such as distress, anxiety and depression in relation to cancer or genetic testing, (2) cognitive outcomes such as knowledge about cancer and genetics, risk perception, or decision making, (3) behavioural outcomes related to screening, surveillance, and genetic testing.

In the absence of a systematic review, it is difficult to distil the vast amount of publications looking at rather diverse psychosocial interventions targeting various psychological, familial or social aspects. The present study aims to systematically map out the available psychosocial interventions for individuals with a family history of CRC and the current state of the research, in order to identify possible gaps and discuss the potential impact of the interventions.

## Methods

An extensive electronic search was conducted to investigate the literature published until June 2020. PubMed, PsycInfo, and Cochrane databases were searched using the following keywords: colon cancer, colorectal cancer, bowel cancer, psychological intervention, psychosocial intervention, counselling, genetic counselling, psychoeducation, psychotherapy. The complete search syntax is presented in Table 1. Reference lists of the articles from the full text assessment phase were manually searched to identify additional studies.

**Table 1:** Search syntax

((((((((((((((((((colon cancer and psychological intervention)) OR (colon cancer and psychosocial intervention)) OR (colon cancer and psychotherapy)) OR (colon cancer and psychoeducational intervention)) OR (colon cancer and counseling)) OR (colon cancer and counselling)) OR (colorectal cancer and psychological intervention)) OR (colorectal cancer and psychosocial intervention)) OR (colorectal cancer and psychotherapy)) OR (colorectal cancer and psychoeducational intervention)) OR (colorectal cancer and counseling)) OR (colorectal cancer and counselling)) OR (bowel cancer and psychological intervention)) OR (bowel cancer and psychosocial intervention)) OR (bowel cancer and psychotherapy)) OR (bowel cancer and psychoeducational intervention)) OR (bowel cancer and counseling)) OR (bowel cancer and counselling)	

Inclusion criteria consisted of (1) quantitative studies published in English that (2) explored the impact of psychosocial interventions for familial CRC, (3) clearly defined the psychosocial intervention offered, and (4) included participants with a family history of CRC. Studies were coded to identify: authors, year of publication, intervention type (genetic counselling, educational intervention, psychological intervention), study design (prospective, experimental), diagnosis (Lynch Syndrome, Familial Adenomatous Polyposis, familial Colorectal Cancer), cancer history (familial, personal), outcome types (affective, behavioural, cognitive, quality of life), providers’ background (genetic counsellor, medical genetics background, non-genetics medical background), intervention format (face-to-face, written, telephone), sample size and mean age of the participants. Two authors independently assessed the studies and extracted the relevant data.

## Results

**Fig. 1:**
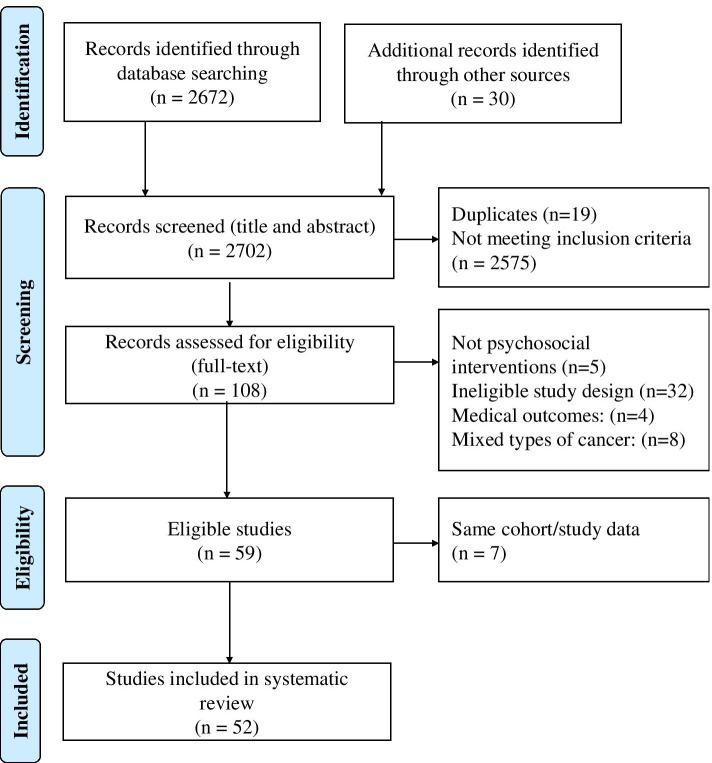
Flow diagram

The literature search yielded 2702 articles. Based on the inclusion criteria, 59 publications were eligible for analysis. Of these, 7 were excluded due to multiple publications from the same cohort [4–10] (e.g. follow-up studies were available and data was more robust in the most recently published article or articles included secondary analyses). The quantitative analysis included 52 articles. Figure 1 shows the literature search flow diagram. The total number of participants included in the studies was 8643; of these, several participants are duplicates due to studies recruiting individuals from the same cohort but provided different interventions and/or measured different outcomes. Table 2. presents the coding and characteristics of the articles included in the review.

**Table 2:** Studies characteristics

No.	Authors, Publication Year	Intervention Type	Study Design	Diagnosis	Cancer History	Outcome type	Provider’s Background	Intervention Format	Mean Age	N
**1**	[11] Aktan-Collan et al., 2007	GC	Prospective	LS	Familial	A, C, QOL	GB	FTF	51,6	72
**2**	[12] Aktan-Collan et al., 2013	GC	Prospective	LS	Familial	A, C, QOL	GB	FTF	44,3	208
**3**	[13] Anderson et al., 2014	EDU	Experimental	fCRC	Familial	A, C	CG	TEL	51,2	272
**4**	[14] Armelao et al., 2010	EDU	Experimental	fCRC	Familial	B	NGB	FTF	57,57	796
**5**	[15] Arver et al., 2004	GC	Prospective	LS	Familial	A, QOL	GB	FTF	42,7	20
**6**	[16] Baghianimoghadam et al., 2012	EDU	Prospective	fCRC	Familial	C	NGB	FTF	39,05	99
**7**	[17] Bastani et al., 2015	EDU	Experimental	fCRC	Familial	A, B	Print/NGB	WRT, TEL	51	1030
**8**	[18] Bauer et al., 2018	EDU/EDU, PSI	Experimental	fCRC	Familial	B	Print/NGB	WRT, TEL	50.8	261
**9**	[19] Brain et al., 2005	GC	Experimental	LS	Familial	A, C, QOL	GB	FTF	41	26
**10**	[20] Burton-Chase et al., 2013	GC	Prospective	LS	Familial	B, C	GC, NBG	FTF	42	78
**11**	[21] Claes et al., 2005	GC, PSI	Prospective	LS	Familial	A	GB, NGB	FTF	39,25	36
**12**	[22] Codori et al., 2003	EDU	Prospective	FAP	Familial	A, B	GB	FTF	11,8	35
**13**	[23] Codori et al., 2005	GC	Prospective	LS	Familial	A, C	GC, NGB	FTF	43,8	101
**14**	[24] Collins et al., 2000 (a)	GC, EDU	Prospective	LS, fCRC	Mixt	C	GC, GB, NGB	FTF	46,7	126
**15**	[25] Collins et al., 2000 (b)	GC, EDU	Prospective	LS, fCRC	Mixt	A	GC, GB, NGB	FTF	47	127
**16**	[26] Collins et al., 2005	GC, EDU	Prospective	LS	Familial	B	NS	FTF	41,33	114
**17**	[27] Collins et al., 2007	GC, EDU	Prospective	LS	Familial	A	NS	FTF	41	73
**18**	[28] Dudok deWit et al., 1998	GC	Prospective	FAP	Familial	A	NGB	FTF	28,6	23
**19**	[29] Esplen et al., 2019	EDU, PSI	Experimental	fCRC	Familial	B, C	GC/NBG	FTF/TEL	47.4	278
**20**	[30] Glanz et al., 2007	EDU	Experimental	fCRC	Familial	A, B, C, QOL	NGB	FTF, TEL	54,4	176
**21**	[31] Gritz et al., 1999	GC	Prospective	LS	Familial	A	GC, NGB	FTF	ns	11
**22**	[32] Gritz et al., 2005	GC	Prospective	LS	Mixt	A, C, QOL	GC, NGB	FTF	ns	155
**23**	[33] Hadley et al., 2004	GC	Prospective	LS	Familial	B	GC	FTF	38,1	56
**24**	[34] Hadley et al., 2008	GC	Prospective	LS	Mixt	C, B	GC	FTF	37	65
**25**	[35] Hadley et al., 2011	GC	Prospective	LS	Mixt	A, B	GC	FTF	41	129
**26**	[36] Halbert et al., 2004	GC	Prospective	LS	Familial	B	NGB	FTF	49,3	71
**27**	[37] Hasenbring et al., 2011	GC	Prospective	LS	Mixt	A	GB, NGB	FTF	40,86	122
**28**	[38] Hawkes et al., 2012	PSI	Prospective	fCRC	Familial	A, B, QOL	NGB	TEL	47,3	22
**29**	[39] Ho et al., 2012	PSI	Prospective	LS, FAP	Mixt	A, C	NGB	FTF	49,4	22
**30**	[40] Ingrand et al., 2016	EDU	Experimental	fCRC	Mixt	B	Print/NGB	WRT, TEL	53,1	429
**31**	[41] Johnson et al., 2002	GC	Prospective	LS, FAP	Familial	B	NS	FTF	55	65
**32**	[42] Keller et al., 2002	GC, PSI, EDU	Prospective	LS	Mixt	A, C, QOL	GB, NGB	FTF	43,29	65
**33**	[43] Keller et al., 2008	GC, PSI, EDU	Prospective	LS	Mixt	A, C	GB, NGB	FTF	44	372
**34**	[44] Kinney et al., 2014	PSI	Experimental	fCRC	Familial	B	Print/GC	WRT, TEL	50,3	378
**35**	[45] Loader et al., 2005	GC	Prospective	LS	Mixt	A, C, B	GC	FTF	59,9	38
**36**	[46] Lowery et al., 2014	EDU/EDU, PSI	Experimental	LS, fCRC	Familial	B, C	Print/NGB	WRT, TEL	ns	632
**37**	[47] Lynch et al., 1997	GC	Prospective	LS	Familial	C	GC	FTF	ns	20
**38**	[48] Manne et al., 2009	EDU/EDU, PSI	Experimental	fCRC	Familial	B, C	Print/NGB	WRT, TEL	47,9	366
**39**	[49] Manne et al., 2010	EDU	Experimental	fCRC	Mixt	A, C, B, QOL	NGB	FTF, WRT	46,3	213
**40**	[50] McClish et al., 2014	EDU	Prospective	fCRC	Familial	B	Print, NS	WRT, TEL	46,8	70
**41**	[51] McGowan et al., 2012	EDU	Experimental	fCRC	Familial	B, C	NGB	FTF	45,5	140
**42**	[52] Meiser et al., 2004	GC	Prospective	LS	Familial	A	NS	FTF	41,3	114
**43**	[53] Murakami et al., 2004	GC	Prospective	LS	Mixt	A	GB	FTF	47	42
**44**	[54] Pieterse et al., 2005	GC	Prospective	fCRC	Mixt	A, C	GB	FTF	48,61	52
**45**	[55] Rawl et al., 2008	EDU	Experimental	fCRC	Familial	B, C	Print	WRT	53	140
**46**	[56] Rawl et al., 2015	EDU	Experimental	fCRC	Familial	B, C	Print/NGB	WRT, TEL	60	145
**47**	[57] Rimes et al., 2006	GC	Prospective	fCRC	Familial	A, C	GB	FTF	44,2	37
**48**	[58] Salimzadeh et al., 2018	PSI	Experimental	fCRC	Familial	B, C	NGB	TEL	47.2	240
**49**	[59] Shiloh et al., 2008	GC	Prospective	LS	Mixt	A	GC	FTF	42,45	253
**50**	[60] Stehpens & Moore, 2007	EDU	Experimental	fCRC	Mixt	B, C	Print	WRT	50,76	91
**51**	[61] Voorwinden & Jaspers, 2015	GC	Prospective	LS	Familial	A, C	GC	FTF	41,87	28
**52**	[62] Wakefield et al., 2008	GC, EDU	Experimental	LS	Mixt	B, C, QOL	Print/NS	FTF, WRT	50,5	109

Intervention: *GC* Genetic counselling; *EDU* Educational interventions; *PSI* psychological interventions; Diagnosis: *LS* Lynch Syndrome, *FAP* Familial Adenomatous Polyposis, *fCRC* familial Colorectal Cancer; Cancer History: Familial, *Mixt* Personal + Familial; Outcome type: *E* emotional; *C* cognitive; *B* behavioural, *QOL* quality of life; Provider’s Background: *GC* genetic counsellor, *GB* medical genetics background, *NGB* non-genetics medical background; Intervention format: *FTF* face to face, *TEL* telephone, *WRT* written, *NS* not specified

## Overview of findings

**Fig. 2:**
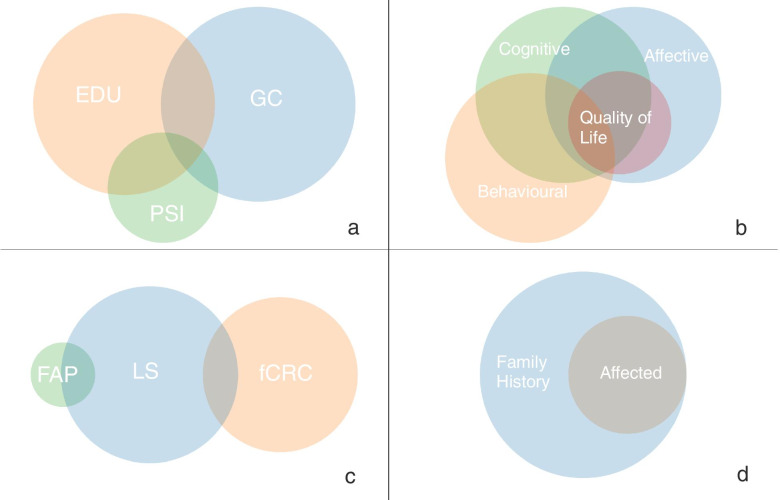
Venn diagrams

Three main types of psychosocial interventions were identified: genetic counselling, educational interventions, psychological interventions; for the purpose of this review, we categorised the various combinations of genetic counselling, educational, and psychological interventions as multimodal interventions. Figure 2a. presents the scaled Venn diagram of the interventions and their intersection represents the multimodal interventions. In terms of explored outcomes, we identified a wide range of affective, cognitive and behavioural outcomes either as a unique, stand-alone measure or in different combinations. Quality of life was one of the explored outcomes, but only in combinations with others. Figure 2b. shows the scaled Venn diagram of the explored outcomes and the intersections represent the different combinations found in the studies. In terms of diagnoses, LS was found in 25 studies, FAP in 2 studies, fCRC in 20 studies and combinations of the three were found in 5 studies. Figure 2c. presents the scaled Venn diagram of the diagnoses and the intersections represent different combinations found in the studies. Individuals with a family history of CRC were included in 35 studies and individuals with both personal and familial history of CRC were included in 17 studies. Figure 2d. presents the scaled Venn diagram of individuals included in the studies based on their familial and personal history of CRC.

### Genetic counselling

Genetic counselling is the process of “helping people understand and adapt to the medical, psychological and familial implications of genetic contributions to disease. This process integrates the following: (1) interpretation of family and medical histories to assess the chance of disease occurrence or recurrence; (2) education about inheritance, testing, management, prevention, resources and research; (3) counselling to promote informed choices and adaptation to the risk or condition” [63]. Genetic counselling was investigated in 23 studies, almost half of the articles included in this review. In 15 studies, it was offered to unaffected family members at risk for CRC, and in 8 studies to individuals with a personal history of CRC. Affective outcomes (e.g. anxiety, depression, emotional distress, and specific fears) were investigated in 17 studies and they represent the most frequently explored outcome. Several studies [12, 27] reported an increase of the emotional distress immediately after a genetic test disclosure session; at follow up the reported scores tended to decrease back to baseline. Cognitive outcomes (e.g. knowledge about CRC and genetics, and perception of risk) were assessed in 12 studies. Behavioural outcomes (e.g. uptake of colonoscopy and gynaecological cancer screening) were addressed in 7 studies and quality of life in 5 studies. Genetic counselling was reported to have a positive impact on screening adherence for the mutation carriers, but non-carriers appeared to comply less with the screening recommendations. Genetic counselling was provided by a genetic counsellor in 11 studies and by a medical professional with background in genetics in 8 studies. In all studies genetic counselling was done face to face and it was usually supplemented by letters after the session. All but one study measured the impact of genetic counselling with a prospective design (e.g., baseline and post intervention questionnaires, without a control group).

### Educational interventions

Educational interventions in cancer setting are aimed at providing information to insure sufficient knowledge about the condition, prevention, management of symptoms. Educational interventions were found in 16 studies, approximately a third of the articles analysed and were mostly focused on providing knowledge about the risk of developing CRC and prevention strategies such as diet, physical activity and screening. In 13 studies, educational interventions were offered to individuals with a family history of CRC. The majority of the educational interventions were offered to individuals with a personal or a family history of fCRC. Behavioural outcomes (e.g. uptake of CRC screening, diet, physical activity) were measured in 12 studies and represent the most frequently investigated outcome. Cognitive outcomes (e.g., knowledge, perceived severity, attitudes towards CRC, screening intention) were explored in 6 studies. Affective outcomes (e.g. anxiety, specific fears, depression, optimism) were explored in 5 studies. Educational interventions were mostly provided by health professionals without a background in genetics (11 studies). The model of delivery was the most diverse across all psychosocial interventions, using written (i.e., booklets, leaflets, CDs), telephone, face to face, and mixed methods. All studies were strongly supportive of the important role education has on screening uptake and reported positive results. The impact of educational interventions on affective outcomes was found to be less prominent.

### Psychological interventions

The psychological interventions found in the studies were based on various psychotherapy paradigms such as acceptance and commitment therapy or motivational interviews, and were aimed at supporting positive life changes, improving uptake of screening, or alleviating emotional distress. Psychological interventions were found in a small proportion of studies (4 studies) and targeted affective (e.g., anxiety, depression, hope), behavioural (e.g., uptake of colonoscopy, food consumption and physical activity) and cognitive outcomes (e.g., knowledge). Three studies included unaffected individuals at risk for fCRC and one study included individuals with a familial history of LS or FAP. The intervention was offered by health professionals with various professional backgrounds such as oncology nursing, clinical psychology, surgery in 3 studies and by a genetic counsellor in 1 study. Psychological interventions were provided by telephone in 3 studies and face to face in 1 study. All studies exploring psychological interventions reported a positive impact in alleviating emotional distress.

### Multimodal interventions

Multimodal interventions consist of different combinations of the 3 main psychosocial interventions, and were explored in 12 studies. The outcomes investigated were varied, including affective outcomes in 5 studies, cognitive outcomes in 7 studies, behavioural outcomes in 6 studies and quality of life in 2 studies. Six studies included participants with a family history of CRC and 6 included participants with both family and personal CRC history. Multimodal interventions were provided face to face, by professionals with a wide variety of backgrounds. Three studies compared a multimodal intervention with educational intervention therefore these studies are included in both categories. All studies providing multimodal interventions predominantly reported positive impact across all types of outcomes measured.

## Discussions

Our analysis provides an overview of the literature exploring the impact of psychosocial interventions for familial CRC. The analysis suggests that psychosocial interventions - genetic counselling, educational and psychological interventions - have an overall positive impact on emotional, cognitive, and behavioural outcomes. With an overview of the research available, we were also able to identify several research gaps and suggest potential strategies to address them.

Although psychosocial interventions generally reported a positive impact, it is essential for future research studies to rigorously assess their efficacy. Results from genetic counselling studies are undoubtedly positive: genetic counselling improves knowledge, emotional distress and screening adherence. In order to provide unequivocal empirical evidence supporting the efficacy of genetic counselling, it is essential for future research to encourage randomised clinical trials. Future research would also benefit from aligning in a more systematic manner the context and content of the interventions with the assessed outcomes. For instance, as hypothesised, educational interventions reported positive results on screening uptake. Yet, unsurprisingly, given the informative nature of the education interventions, their impact on affective outcomes was less prominent. This is in line with previous research in genetic counselling [64] and substantial empirical evidence from clinical psychology [65] showing that knowledge does not necessarily alleviate emotional distress. Undoubtedly, there is a clear need for more studies exploring the impact of psychological interventions for familial CRC. Psychological interventions have a strong empirical evidence base supporting their benefit in alleviating emotional distress for cancer in general [66], and various medical conditions [67], therefore only identifying 4 studies investigating psychological interventions was surprising. Although valuable in themselves, future research exploring multimodal interventions would also benefit from more clarity regarding the theory underlying the various psychosocial interventions, the expected mechanisms of change of the interventions offered and the specificity of the outcome measures used. That said, given the heterogeneity of the multimodal interventions, the rather modest impact reported was perhaps not surprising.

To conclude, the increased number of studies exploring psychosocial interventions for CRC and the positive impact reported was indeed encouraging. Mapping this research area also highlighted several limitations of the research in this field. The heterogeneity of the research designs, outcomes and measures used could benefit from a more programmatic approach. In order for psychosocial interventions to gather a critical mass of empirical evidence, to support their efficacy and clarify their mechanisms of change, robust research studies need to be designed and implemented.

## Data Availability

All data generated or analysed during this study are included in this published article and its supplementary information file.
